# The Role of Patient Activation in Preferences for Shared Decision Making: Results From a National Survey of U.S. Adults

**DOI:** 10.1080/10810730.2015.1033115

**Published:** 2015-08-27

**Authors:** Samuel G. Smith, Anjali Pandit, Steven R. Rush, Michael S. Wolf, Carol J. Simon

**Affiliations:** ^a^ Division of General Internal Medicine, Feinberg School of Medicine, Northwestern University, Chicago, Illinois, USA; ^b^ Wolfson Institute of Preventive Medicine, Queen Mary University of London, London, United Kingdom; ^c^ United Healthcare, Minneapolis, Minnesota, USA; ^d^ Optum Institute, Minneapolis, Minnesota, USA

## Abstract

Studies investigating preferences for shared decision making (SDM) have focused on associations with sociodemographic variables, with few investigations exploring patient factors. We aimed to investigate the relationship between patient activation and preferences for SDM in 6 common medical decisions among a nationally representative cross-sectional survey of American adults. Adults older than 18 were recruited online (*n* = 2,700) and by telephone (*n* = 700). Respondents completed sociodemographic assessments and the Patient Activation Measure. They were also asked whether they perceived benefit (yes/no) in SDM in 6 common medical decisions. Nearly half of the sample (45.9%) reached the highest level of activation (Level 4). Activation was associated with age (*p* < .001), higher income (*p* = .001), higher education (*p* = .010), better self-rated health (*p* < .001), and fewer chronic conditions (*p* = .050). The proportion of people who agreed that SDM was beneficial varied from 53.1% (deciding the necessity of a diagnostic test) to 71.8% (decisions associated with making lifestyle changes). After we controlled for participant characteristics, higher activation was associated with greater perceived benefit in SDM across 4 of the 6 decisions. Preferences for SDM varied among 6 common medical scenarios. Low patient activation is an important barrier to SDM that could be ameliorated through the development of behavioral interventions.

The health care community is increasingly embracing shared decision making (SDM; Kon, 2010), with several large organizations and patient groups recommending it as the preferred communicative approach in medical care (Davidson et al., 2007; Institute of Medicine, 2001; Mercurio, Maxwell, Mears, Ross, & Silber, 2008; Sheridan, Harris, & Woolf, 2004). These positions are supported by ethical principles and federal and state laws (Centers for Medicare and Medicaid Services, 2011; Kuehn, 2009). Although the definitions of SDM are contested, clarifying and including patient preferences within the medical decision-making process and discussing all available treatment options are generally accepted components (Makoul & Clayman, 2006). SDM can lead to improved decision making (Krones et al., 2008; McCaffery, Smith, & Wolf, 2010; Stacey et al., 2014; Zikmund-Fisher, Couper, Singer, Ubel, et al., 2010) and reduced health care costs (Arterburn et al., 2012; Veroff, Marr, & Wennberg, 2013; Wennberg, Marr, Lang, O'Malley, & Bennett, 2010). The relationship between SDM and health outcomes is mixed (Joosten et al., 2008; Stacey et al., 2014; Street, Makoul, Arora, & Epstein, 2009; Ward et al., 2003; Wilson et al., 2010).

The nationally representative DECISIONS survey found that the majority (80%) of Americans had made a medical decision in the previous 2 years (Zikmund-Fisher, Couper, Singer, Levin, et al., 2010). However, only 40% to 65% of the decisions were shared between the patient and the provider (Zikmund-Fisher, Couper, Singer, Ubel, et al., 2010). Preferences for involvement in medical decision making can vary according to the situation (Say, Murtagh, & Thomson, 2006). For example, members of the public express more interest in being involved in disease prevention decisions such as smoking cessation and lifestyle change (Mansell, Poses, Kazis, & Duefield, 2000) but prefer to be more passive in situations with more immediate consequences (e.g., cancer treatment; Chewning et al., 2012; Davis, Hoffman, & Hsu, 1999). Yet these generalizations are not universal, and research investigating variation across decision types is needed (Mansell et al., 2000; Waller et al., 2012).

Early studies showed that patient characteristics such as male gender, worse self-reported health, and lower socioeconomic status predicted less preference for SDM (Arora & McHorney, 2000; Kiesler & Auerbach, 2006; Say et al., 2006). However, these studies often used small samples from specific clinical populations, which can limit generalizations. A limited number of nationally representative studies are available (Levinson, Kao, Kuby, & Thisted, 2005; Zikmund-Fisher, Couper, Singer, Ubel, et al., 2010); however, additional data are needed that go beyond associations with sociodemographic factors, which have explained comparatively little variance in preferences for SDM (Say et al., 2006). SDM requires the patient to recognize his or her own values and incorporate them within the decision-making process. Thus, psychological factors such as motivation and engagement in a health and medical context may be important concepts to study. To a large extent, patient activation encapsulates these factors; it refers to the degree to which an individual possesses knowledge, motivation, skills, and confidence to make effective health-related decisions (Hibbard, Mahoney, Stockard, & Tusler, 2005). Activated patients have better physical and mental health (Smith, Curtis, Wardle, von Wagner, & Wolf, 2013), more optimal clinical indicators (Greene & Hibbard, 2012; Marshall et al., 2013), and lower health care costs (Greene & Hibbard, 2012). Disparities in activation exist across economic and ethnic groups (Greene & Hibbard, 2012; Hibbard & Cunningham, 2008), and simulated models indicate that meaningful reductions in health inequalities could occur if activation differences were eliminated (Hibbard et al., 2008).

The relationship between activation and SDM preferences is underinvestigated, but preliminary evidence suggests that activating patients can increase SDM (Wilkes et al., 2013). Activated patients seek more health-related information and are more knowledgeable about health care (Butler, Farley, Sleath, Murray, & Maciejewski, 2012; Fowles et al., 2009; Harvey, Fowles, Xi, & Terry, 2012). They may therefore be better prepared prior to consultations and show greater preferences for involvement in medical decision making. To progress the field and inform future intervention strategies, we used nationally representative data to investigate the role of patient activation and sociodemographic factors within preferences for SDM across six common medical decisions. Based on previous evidence, we hypothesized that (a) preferences for SDM would be higher in situations involving disease prevention (e.g., screening and lifestyle decisions) and lower when the consequences are more immediate/important (e.g., diagnostic testing and treatment); (b) higher patient activation would be associated with greater perceived benefit in SDM; (c) members of lower socioeconomic groups (as measured by income, education, and race), men, younger respondents, and those with worse health would have lower levels of activation; and (d) members of lower socioeconomic groups, younger respondents, and those with better health would perceive less benefit in SDM.

## Methods

### Design

Data were from a nationally representative survey of English-speaking U.S. adults age 18 years and older. The survey was conducted by Harris Interactive between August 14 and September 17, 2013. To ensure full representation of U.S. adults, a mixed-mode methodology was used that combined an online panel (*n* = 2,700) and random-digit dialing (RDD; *n* = 700) sampling and data collection. The telephone sample included landline and cell phone connections and was randomized prior to dialing. For the online sample, a consumer research panel associated with Harris Interactive was stratified by U.S. Census parameters (education, age by gender, race, region, and household income) and then invited by e-mail to take part in a self-administered survey (U.S. Census Bureau, 2012). The e-mail invitation included a secure and unique URL and password to ensure that the respondent could only complete one survey. Excluding disconnected lines, a total of 15,050 working phone numbers yielded 838 participating respondents, for an overall response rate of 5.6% in the RDD sample. Of the 838 respondents who were screened, 700 met the age qualification (U.S. resident, age 18 or older) specified in the study design. A response rate for the online sample is not reported as probability sampling was not used.

In accordance with polling industry standards, respondents' confidentiality was ensured such that they were not identified by name or any other personal identifiers. As a member of the Council of American Survey Research Organizations (CASRO), the research company conducted this survey in accordance with the CASRO Code of Standards and Ethics for Survey Research.

### Measures

#### SDM

To assess preferences for patient involvement in SDM, a series of common medical decisions were presented to respondents with the following introductory statement:

There has been much discussion lately about patients participating with their healthcare providers in making decisions about their health. For which of these topic areas do you think it is beneficial for patients to share in decisions with their healthcare provider?

The following medical decisions were provided: lifestyle changes, such as diet and exercise; preventive screening tests; choosing between completely different approaches to treating a diagnosed condition (e.g., medication vs. surgery); choosing a specialist for referral; choosing between different medications that might be prescribed for a diagnosed condition; and deciding whether it is necessary to have a diagnostic test. The response items were *yes* and *no.* To prevent ordering effects, the decisions were presented in a random order for each participant.

#### Patient Activation Measure

Patient activation was assessed using the Patient Activation Measure Short Form (Hibbard et al., 2005). This is a 13-item measure designed to assess self-reported knowledge, skill, and confidence for self-management of one's health and chronic conditions. The measure has been shown to be reliable and valid across a range of clinical conditions and contexts (Hibbard, Stockard, Mahoney, & Tusler, 2004; Hung et al., 2013; Skolasky et al., 2011). Participants were asked to respond to statements that people sometimes make when they talk about their health. Example statements included “When all is said and done, I am the person who is responsible for managing my health condition(s)” and “I am confident that I can take actions that will help prevent or minimize some symptoms or problems associated with my health condition(s).” Response items included *strongly disagree, slightly disagree, slightly agree, strongly agree, not applicable, don't know,* and *refused.* A score of 0 (low activation) to 100 (high activation) was computed, and respondents were classified into four levels of activation using predefined cutoffs: Level 1—may not yet believe that the patient role is important (0–45.2), Level 2—lacks confidence and knowledge to take action (47.4–52.9), Level 3—beginning to take action (56.4–66.0), Level 4—has difficulty maintaining behaviors over time (68.5–100). The measure had good internal reliability in this sample (α = .90).

#### Participant Characteristics

Data on gender, age, race (White, Black, Asian or Pacific Islander, other), income (<$15,000, $15,000–$34,999, $35,000–$49,999, $50,000+), education (high school or less, some college, college graduate, graduate school), region (East, Midwest, South, West), self-rated health (poor, fair, good, very good, excellent), and self-reported chronic conditions (0, 1, or 2+) were collected.

### Statistical Analysis

RDD data were weighted to U.S. Census targets for education, age by gender, race, region, and household income to bring them into line with the population of U.S. residents age 18 and older (U.S. Census Bureau, 2012). The weighting algorithm also took into account landline versus cell phone usage using information from the 2012 National Health Interview Survey (Blumberg & Luke, 2012) and probability of selection using number of telephone lines, number of adults in the household, and recent absence of a phone connection. The online data were also weighted to the same applicable parameters as the telephone RDD sample. However, the algorithm included a propensity score that allowed for adjustment for attitudinal and behavioral differences between online and offline populations, those who join online panels, and likelihood of response. The RDD and online sample data were combined into a proportioned total using Internet usage information (Pew Research Center, 2013).

Complex samples analyses using the weighting variable were performed using the following statistical tests. Chi-square analyses explored which participant characteristics were associated with patient activation. Odds ratios (ORs) and 95% confidence intervals (CIs) for the associations between patient activation, participant characteristics, and perceived benefit in SDM in each decision were computed using multivariate logistic regression. Significance was set at *p* < .05, and all analyses were performed in SPSS Version 22.

## Results

### Sample Characteristics

As shown in Table 1, the weighted analyses showed that respondents were more likely to be female (52.8%), younger (18–30 = 21.9%, 31–40 = 16.6%), White (77.5%), and from the southern United States (33.4%). Half (50.0%) of the respondents had an income >$50,000, and more than a quarter (28.0%) had at least a university-level education. Self-reported health was high (good = 35.9%, very good = 33.4%, excellent = 14.1%), and the majority reported no chronic conditions (66.5%). In line with the recruitment strategy, most people completed the survey online (77.0%).

**Table 1.** Participant characteristics and percentage at highest level of activation

Characteristic	*n*	%	Level 1	Level 2	Level 3	Level 4	χ^2^ significance
Gender							
Male	1,382	47.2	12.4	12.1	30.3	45.2	.400
Female	2,018	52.8	10.2	13.8	29.4	46.6	
Age							
18–30	585	21.9	16.4	12.3	26.2	45.0	.000
31–40	537	16.6	7.6	11.3	34.2	47.0	
41–50	608	18.5	14.4	11.5	29.3	44.9	
51–60	649	17.3	8.9	12.2	33.5	45.4	
61–70	656	16.1	7.4	12.7	26.2	53.7	
71–80	281	7.3	12.1	19.6	30.9	37.4	
80+	84	2.4	7.1	28.9	32.1	32.0	
Ethnicity							
White	2,717	77.5	10.0	12.8	30.5	46.7	.149
Black	346	11.4	12.9	10.5	27.2	49.5	
Asian or Pacific Islander	101	2.2	18.1	18.4	31.7	31.7	
Other	199	8.9	16.4	16.4	28.2	39.0	
Income							
<$15,000	396	9.7	15.1	16.5	30.0	38.4	.001
$15,000–$34,999	773	18.3	13.1	15.5	30.2	41.2	
$35,000–$49,999	449	12.9	14.0	14.7	31.7	39.6	
>$50,000	1,500	50.0	8.4	10.6	28.8	52.1	
Missing	282	9.2	15.1	14.7	31.8	38.4	
Education							
High school or less	884	43.2	13.9	13.9	29.8	42.3	.010
Some college	1,202	28.9	9.8	12.6	32.7	45.0	
College graduate	886	20.4	8.4	12.3	27.4	51.9	
Graduate school	411	7.6	10.2	11.1	23.9	54.8	
Region							
East	764	21.5	10.3	12.5	31.6	45.5	.804
Midwest	833	22.4	11.3	12.4	31.8	44.5	
South	1,041	33.4	10.6	12.5	29.0	47.9	
West	762	22.7	13.0	14.8	27.3	44.8	
Survey mode							
Online	2,700	77.0	10.8	12.5	29.7	47.1	.317
Phone	700	23.0	12.9	14.7	30.3	42.1	
Self-rated health							
Poor	132	3.6	27.6	17.5	20.3	34.6	.000
Fair	514	13.0	22.8	18.0	29.2	29.9	
Good	1,324	35.9	13.0	16.5	34.7	35.8	
Very good	1,042	33.4	5.4	9.4	30.4	54.7	
Excellent	388	14.1	6.2	6.9	19.3	67.5	
Chronic conditions							
0	983	33.5	7.9	11.9	32.1	48.1	.050
1	816	24.3	11.2	13.4	30.1	45.2	
2+	1,601	42.2	13.8	13.5	28.0	44.7	

*Note. n* may not round to 3,400 because of missing data; percentages are weighted.

### Patient Activation

The mean level of patient activation was 66.6 (*SD* = 16.8). There were fewer people in the lower levels of patient activation (Level 1 = 11.3%, Level 2 = 13.0%, Level 3 = 29.8%), with nearly half (45.9%) of the sample reaching the highest level of activation (Level 4). There was a relationship between patient activation and age (*p* < .001). Respondents age 61–70 were most likely to be in the highest level of activation (53.7%), and the youngest and oldest age groups were less likely (see Table 1). Income (*p* = .001) and education (*p* = .010) were significantly associated with patient activation. The highest levels of activation were observed in those with the highest income ($50,000 + = 52.1%) and highest education (graduate school = 54.8%). Asians or Pacific Islanders (31.7%) or the “other” race category (39.0%) had notably lower levels of activation, but there was no overall effect of race (*p* = .149). Respondents with better self-rated health (*p* < .001) and fewer chronic conditions (*p* = .050) were more likely to have higher activation. Activation levels were similar between genders (*p* = .400), regions (*p* = .804), and survey modes (*p* = .317).

### SDM

Respondents generally indicated that they saw benefit in SDM across all medical decisions (see Table 2). The proportion of endorsement ranged from 53.1% (deciding the necessity of a diagnostic test) to 71.8% (decisions regarding lifestyle changes). Table 2 describes the relationship between SDM and patient activation in univariate analyses for each of the six decisions. Differences were observed for lifestyle (*p* < .001), preventive screening (*p* < .001), choosing treatments (*p* = .012), choosing between medications (*p* = .018), and deciding the necessity of a diagnostic test (*p* = .021). There were no differences across activation groups for the decision related to choosing a specialist for referral (*p* = .804).

**Table 2.** Likelihood of perceived benefit in shared decision making by decision type and activation level

Decision type	Overall (%)	Level 1 (%)	Level 2 (%)	Level 3 (%)	Level 4 (%)	χ^2^
Lifestyle change	71.8	62.6	70.5	71.5	77.8	.000
Preventive screening tests	59.2	47.6	59.7	59.1	64.6	.000
Choosing between different treatment approaches	60.1	56.1	58.7	57.7	65.7	.012
Choosing a specialist for referral	54.7	52.7	56.0	56.9	55.6	.804
Choosing between different medications	58.0	49.7	58.6	58.1	62.3	.018
Deciding the necessity of a diagnostic test	53.1	50.2	50.1	51.3	58.5	.021

In multivariate analyses, patient activation was associated with perceived benefit in SDM in four of the six decisions (see Table 3). Compared with the reference category (low activation, Level 1), respondents with the highest level of activation (Level 4) were more likely to see benefit in SDM for decisions related to lifestyle (OR = 2.13, 95% CI [1.43, 3.18]), preventive screening (OR = 2.02, 95% CI [1.41, 2.91]), choosing treatments (OR = 1.50, 95% CI [1.00, 2.13]), and choosing medications (OR = 1.82, 95% CI [1.25, 2.66]). Respondents in Level 3 activation were more likely to see benefit in SDM for decisions related to preventive screening (OR = 1.65, 95% CI [1.14, 2.38]) and choosing medications (OR = 1.53, 95% CI [1.04, 2.24]). Respondents in Level 2 activation were only more likely to see benefit for the preventive screening decision (OR = 1.62, 95% CI [1.08, 2.44]).

**Table 3.** Weighted multivariate logistic regression predicting a “yes” response to seeing perceived benefit in shared decision making

Characteristic	Lifestyle	Preventive screening	Choosing treatments	Choosing specialists	Choosing medications	Diagnostic testing
	OR [95% CI]	OR [95% CI]	OR [95% CI]	OR [95% CI]	OR [95% CI]	OR [95% CI]
Gender						
Male	Ref	Ref	Ref	Ref	Ref	Ref
Female	1.21 [0.97, 1.53]	1.10 [0.90, 1.36]	1.44 [1.17, 1.78]	1.46 [1.19, 1.79]	1.18 [0.96, 1.44]	1.14 [0.93, 1.39]
Age						
18–30	Ref	Ref	Ref	Ref	Ref	Ref
31–40	1.08 [0.75, 1.55]	1.30 [0.92, 1.83]	0.95 [0.67, 1.35]	0.97 [0.68, 1.36]	1.02 [0.72, 1.44]	1.12 [0.79, 1.59]
41–50	1.10 [0.77, 1.58]	1.25 [0.89, 1.75]	0.99 [0.71, 1.39]	0.92 [0.66, 1.28]	1.06 [0.76, 1.48]	1.21 [0.87, 1.70]
51–60	2.03 [1.37, 3.00]	2.05 [1.45, 2.89]	1.63 [1.15, 2.30]	1.69 [1.21, 2.36]	1.38 [0.98, 1.93]	1.80 [1.29, 2.52]
61–70	1.65 [1.13, 2.41]	2.06 [1.44, 2.94]	1.49 [1.04, 2.14]	1.81 [1.29, 2.55]	1.35 [0.95, 1.92]	2.09 [1.47, 3.00]
71–80	1.86 [1.07, 3.22]	2.57 [1.57, 4.20]	1.46 [0.93, 2.31]	1.84 [1.16, 2.95]	1.12 [0.71, 1.77]	2.33 [1.49, 3.64]
80+	1.49 [0.70, 3.17]	1.29 [0.69, 2.45]	1.18 [0.61, 2.28]	2.05 [1.02, 4.11]	1.30 [0.67, 2.53]	2.83 [1.44, 5.58]
Ethnicity						
White	Ref	Ref	Ref	Ref	Ref	Ref
Black	0.87 [0.60, 1.26]	0.77 [0.55, 1.07]	0.81 [0.58, 1.13]	1.03 [0.74, 1.43]	0.67 [0.48, 0.93]	0.86 [0.61, 1.21]
Asian or Pacific Islander	0.92 [0.42, 2.00]	0.53 [0.27, 1.05]	0.45 [0.26, 0.81]	0.59 [0.28, 1.23]	0.47 [0.25, 0.89]	0.83 [0.42, 1.63]
Other	1.22 [0.79, 1.88]	1.07 [0.71, 1.62]	1.61 [1.04, 2.50]	1.49 [0.99, 2.23]	1.42 [0.94, 2.13]	1.80 [1.19, 2.72]
Income						
<$15,000	Ref	Ref	Ref	Ref	Ref	Ref
$15,000–$34,999	0.87 [0.58, 1.30]	1.21 [0.83, 1.77]	1.22 [0.84, 1.76]	1.41 [0.98, 2.03]	0.91 [0.63, 1.33]	0.97 [0.67, 1.41]
$35,000–$49,999	1.09 [0.69, 1.73]	1.09 [0.72, 1.66]	1.05 [0.69, 1.59]	1.17 [0.77, 1.78]	0.96 [0.63, 1.45]	0.77 [0.51, 1.18]
$50,000+	0.98 [0.67, 1.45]	1.12 [0.78, 1.61]	0.97 [0.68, 1.39]	1.42 [0.99, 2.04]	0.93 [0.65, 1.34]	0.95 [0.66, 1.37]
Missing	1.43 [0.82, 2.49]	2.16 [1.29, 3.60]	1.14 [0.71, 1.85]	2.59 [1.58, 4.24]	1.36 [0.81, 2.26]	1.43 [0.87, 2.35]
Education						
High school or less	Ref	Ref	Ref	Ref	Ref	Ref
Some college	1.47 [1.12, 1.95]	1.24 [0.96, 1.59]	1.12 [0.87, 1.44]	1.15 [0.89, 1.47]	0.92 [0.71, 1.18]	1.00 [0.78, 1.28]
College graduate	1.70 [1.23, 2.40]	1.60 [1.18, 2.15]	1.63 [1.21, 2.19]	1.24 [0.92, 1.65]	1.03 [0.78, 1.37]	1.02 [0.76, 1.37]
Graduate school	1.99 [1.28, 3.11]	1.57 [1.06, 2.31]	2.54 [1.71, 3.79]	1.57 [1.08, 2.28]	1.17 [0.81, 1.68]	0.92 [0.63, 1.32]
Patient activation						
Level 1	Ref	Ref	Ref	Ref	Ref	Ref
Level 2	1.33 [0.86, 2.08]	1.62 [1.08, 2.44]	1.06 [0.70, 1.60]	1.13 [0.74, 1.70]	1.47 [0.97, 2.22]	0.92 [0.61, 1.38]
Level 3	1.42 [0.95, 2.12]	1.65 [1.14, 2.38]	1.05 [0.71, 1.54]	1.25 [0.86, 1.83]	1.53 [1.04, 2.24]	1.04 [0.71, 1.51]
Level 4	2.13 [1.43, 3.18]	2.02 [1.41, 2.91]	1.50 [1.00, 2.13]	1.17 [0.81, 1.71]	1.82 [1.25, 2.66]	1.31 [0.97, 2.05]
Self-rated health						
Poor	1.35 [0.62, 2.93]	1.80 [0.88, 3.66]	1.39 [0.68, 2.82]	2.93 [1.51, 5.69]	1.51 [0.73, 3.13]	1.95 [0.95, 4.00]
Fair	1.41 [0.88, 2.28]	1.13 [0.73, 1.74]	0.99 [0.64, 1.52]	1.22 [0.81, 1.85]	0.91 [0.59, 1.41]	0.82 [0.53, 1.25]
Good	1.37 [0.93, 2.03]	0.80 [0.56, 1.12]	0.89 [0.62, 1.26]	0.87 [0.62, 1.23]	0.81 [0.57, 1.15]	0.67 [0.48, 0.95]
Very good	1.40 [0.95, 2.05]	0.87 [0.61, 1.23]	1.03 [0.73, 1.45]	1.09 [0.78, 1.52]	0.85 [0.60, 1.19]	0.79 [0.56, 1.11]
Excellent	Ref	Ref	Ref	Ref	Ref	Ref
Chronic conditions						
0	Ref	Ref	Ref	Ref	Ref	Ref
1	0.99 [0.74, 1.34]	1.17 [0.90, 1.55]	1.17 [0.88, 1.54]	0.95 [0.72, 1.25]	1.19 [0.98, 1.69]	0.98 [0.74, 1.29]
2+	1.34 [0.99, 1.82]	1.32 [1.00, 1.72]	1.36 [1.04, 1.79]	1.16 [0.90, 1.51]	1.62 [1.25, 2.11]	1.14 [0.88, 1.49]

*Note.* OR = odds ratio; CI = confidence interval.

To various extents, participant characteristics were associated with SDM preferences. Women were more likely to see benefit in the decisions related to choosing treatment (OR = 1.44, 95% CI [1.17, 1.78]) and choosing a specialist (OR = 1.46, 95% CI [1.19, 1.79]). Older respondents were generally more likely to see benefit in SDM than the youngest participants. Black respondents were less likely to see benefit in decisions related to medication choice (OR = 0.81, 95% CI [0.58, 1.13]), whereas Asian or Pacific Islanders were less likely to see benefit in choosing treatments (OR = 0.45, 95% CI [0.26, 0.81]) and choosing medications (OR = 0.47, 95% CI [0.25, 0.89]). Members of the “other” race category were more likely to see benefit in choosing treatments (OR = 1.61, 95% CI [1.04, 2.50]) and diagnostic testing (OR = 1.80, 95% CI [1.19, 2.72]). Income was rarely a predictor of SDM preferences, with the exception of the “missing” category and decisions related to preventive screening (OR = 2.16, 95% CI [1.29, 3.60]) and choosing a specialist (OR = 2.59, 95% CI [1.58, 4.24]).

Higher education was associated with preferences for SDM. For example, compared with the least educated (high school or less), those who attended graduate school were more likely to perceive benefit in SDM for decisions related to lifestyle (OR = 1.99, 95% CI [1.28, 3.11]), preventive screening (OR = 1.57, 95% CI [1.06, 2.31]), choosing treatments (OR = 2.54, 95% CI [1.71, 3.79]), and choosing specialists (OR = 1.57, 95% CI [1.08, 2.28]). Respondents with college education were more likely to perceive benefit in the decisions related to lifestyle (OR = 1.70, 95% CI [1.23, 2.40]), preventive screening (OR = 1.60, 95% CI [1.18, 2.15]), and choosing treatment (OR = 1.63, 95% CI [1.21, 2.19]).

The impact of health status varied across decisions. Compared with those with excellent self-reported health, respondents with poor self-rated health were more likely to see benefit in SDM when choosing a specialist (OR = 2.93, 95% CI [1.51, 5.69]). Respondents with good health were less likely to see benefit in SDM in diagnostic testing (OR = 0.67, 95% CI [0.56, 1.11]), but there was no discernable effect for fair or very good health. Those with two or more chronic conditions were more likely to see benefit in SDM for decisions related to preventive screening (OR = 1.32, 95% CI [1.00, 1.72]), choosing treatment (OR = 1.36, 95% CI [1.04, 1.79]), and choosing medications (OR = 1.62, 95% CI [1.25, 2.11]).

## Discussion

In this nationally representative survey the majority of U.S. adults perceived at least some benefit in SDM, but there was a sizeable minority who did not. The attitudes of the population should be considered by large organizations and policymakers who are increasingly advocating a position of SDM (Davidson et al., 2007; Institute of Medicine, 2001; Mercurio et al., 2008; Sheridan et al., 2004). Perceived benefit in SDM varied according to the decision. Patients preferred greater involvement in decisions involving disease prevention (e.g., screening) and lifestyle change. Less perceived benefit was reported for decisions involving immediate consequences, such as deciding the necessity of a diagnostic test. However, this assertion did not generalize to decisions involving treatment and medication, for which the consequences may also be expected to be important and immediate. Overall, estimates of interest in SDM broadly reflected actual occurrences of SDM reported in a nationally representative sample (Zikmund-Fisher, Couper, Singer, Ubel, et al., 2010).

Although our study design prohibits inferences of causality, our findings suggest that low levels of patient activation may be a barrier to SDM. Respondents reaching the highest activation level were consistently more likely to perceive benefit in SDM across all medical decisions, with the exception of choosing a specialist. The relationship between activation and perceived benefit in SDM among the lower levels of activation (Levels 2 and 3) was less consistent. For example, compared with the least activated respondents, those classified at Levels 2, 3, or 4 of activation were more likely to see benefit in SDM in the preventive screening decision and in choosing medications. However, for other decisions (e.g., lifestyle changes and choosing medical treatments), it was not until the highest levels of activation were reached that respondents perceived benefit.

Patient activation appeared to affect preferences most in decisions regarding preventive screening and choosing medications, in which even moderately activated respondents preferred greater involvement than those who were least activated. In contrast, decisions involving lifestyle changes and choosing treatments may need patients to increase to the highest activation level before benefits will be seen in terms of SDM preferences. Interventions such as preparing patients prior to consultations through preference elicitation and clarification have been somewhat effective at increasing activation (Deen, Lu, Rothstein, Santana, & Gold, 2011). However, they may not be sufficiently effective among the least activated for improvements to be seen in SDM preferences in some decisions. Decisions involving treatment choices may require alternative multifaceted approaches, such as decision aids plus patient preparation, to promote the necessary amount of activation (Deen et al., 2012; Hibbard, Greene, & Tusler, 2009).

Our data indicated that a little less than half (45.9%) of the population were classified as having Level 4 activation, which is reflective of previous national estimates (Hibbard & Cunningham, 2008). Our data also support evidence showing that patient activation is associated with markers of socioeconomic status, including income and education, and other demographic factors, such as age (Greene & Hibbard, 2012; Hibbard & Cunningham, 2008). Patient activation was higher among healthier individuals, as measured by self-reported health and the number of chronic conditions. To our knowledge, this has not been reported in nationally representative samples, although it has been suggested by research in specific clinical populations (Chubak et al., 2012). In contrast to previous national estimates, there was no overall effect of race on patient activation levels (Hibbard & Cunningham, 2008). However, the prevalence of high activation among Asian and Pacific Islander respondents was noticeably lower than among the other ethnicities. We found no evidence of gender differences, but only small effects were noted in the large sample reported by Hibbard and Cunningham (2008).

In line with previous research, low education was consistently associated with perceiving less benefit in SDM (Arora & McHorney, 2000). One possibility is that people with low levels of education lack the skills to adequately process health information and therefore prefer to rely on expert opinion to make medical decisions. The term *health literacy* generally describes these skills and has been defined by the Institute of Medicine as “the degree to which individuals have the capacity to obtain, process and understand basic health information and services needed to make appropriate health decisions” (Ratzan & Parker, 2000, p. 19). In previous analyses we showed that health literacy and patient activation influenced health outcomes through different pathways, and this may also be the case with SDM (Smith et al., 2013). Future research should consider investigating the importance of these two variables in the formation of SDM preferences.

No effects were consistently observed for income on SDM preferences, perhaps because of confounding with education. Effects by race were observed, with respondents classified as “other” more likely to see benefit in choosing treatments, specialists, and diagnostic testing. Differences across race/ethnicity were also observed for the treatment and medications decisions. In support of previous findings we observed higher preferences for SDM among those who were older (Levinson et al., 2005), but gender effects did not often reach statistical significance. These inconsistencies across conditions further emphasize the importance of specifying the type of decision when asking about SDM preferences. They also highlight areas in which disparities are most likely to occur, providing guidance for prospective interventions.

This study has limitations. Items were phrased to ask whether respondents could “see benefit” in SDM rather than specifically using the word *preference.* The introductory statement to the SDM item had a readability score of 46 out of 100, which is equivalent to a reading age of 16 years. The text may therefore have been difficult for people with lower levels of literacy to comprehend. The study is further limited by the simplicity of the items used. Alternative approaches (e.g., qualitative approaches) may yield a richer data set but would be limited by their low external validity (Edwards & Elwyn, 2006; Elwyn, Edwards, Kinnersley, & Grol, 2000). The decisions presented were hypothetical, and therefore reported preferences may not reflect attitudes held by patients actually making those judgments. Similarly, we were unable to collect data on previous experience with decision making, and it is unclear whether respondents had experience in each scenario. Response and selection biases were limited through weighting procedures, but it is possible that the online panel sample may have been more activated than an offline population. We may therefore have overestimated the level of patient activation in the general U.S. sample, and associations with demographic variables may be conservative estimates. The response rates in our RDD survey were also low in comparison with similar studies (Cantor et al., 2009; Cantor, Covell, Davis, Park, & Rizzo, 2007). Finally, it should be acknowledged that our data were cross-sectional, which limits our ability to assert causality.

## Conclusions

In conclusion, this nationally representative study indicates that there is a discord between policy and public attitudes toward SDM. Although clinicians are encouraged to promote SDM, a large proportion of the public in this study reported not perceiving any benefit in the practice. Patient activation was a consistent predictor of SDM preferences, indicating that intervention strategies targeting the construct may have beneficial effects on public attitudes toward communication and decision making in clinical encounters. Further prospective and experimental research is warranted on the basis of these findings.
